# Bio-inspired design of a self-aligning, lightweight, and highly-compliant cable-driven knee exoskeleton

**DOI:** 10.3389/fnhum.2022.1018160

**Published:** 2022-11-07

**Authors:** Shuangyue Yu, Tzu-Hao Huang, Antonio Di Lallo, Sainan Zhang, Tian Wang, Qiushi Fu, Hao Su

**Affiliations:** ^1^Lab of Biomechatronics and Intelligent Robotics, Department of Mechanical and Aerospace Engineering, North Carolina State University, Raleigh, NC, United States; ^2^Department of Mechanical Engineering, City College of New York, New York, NY, United States; ^3^Mechanical and Aerospace Engineering, University of Central Florida, Orlando, FL, United States; ^4^NeuroMechanical Systems Laboratory, Biionix (Bionic Materials, Implants & Interfaces) Cluster, University of Central Florida, Orlando, FL, United States; ^5^Joint North Carolina State University/The University of North Carolina Department of Biomedical Engineering, NC State University, Raleigh, NC, United States; ^6^Joint North Carolina State University/The University of North Carolina Department of Biomedical Engineering, University of North Carolina at Chapel Hill, Chapel Hill, NC, United States

**Keywords:** bioinspired design, cable-driven, self-alignment, knee exoskeleton, complaint actuators

## Abstract

Powered knee exoskeletons have shown potential for mobility restoration and power augmentation. However, the benefits of exoskeletons are partially offset by some design challenges that still limit their positive effects on people. Among them, joint misalignment is a critical aspect mostly because the human knee joint movement is not a fixed-axis rotation. In addition, remarkable mass and stiffness are also limitations. Aiming to minimize joint misalignment, this paper proposes a bio-inspired knee exoskeleton with a joint design that mimics the human knee joint. Moreover, to accomplish a lightweight and high compliance design, a high stiffness cable-tension amplification mechanism is leveraged. Simulation results indicate our design can reduce 49.3 and 71.9% maximum total misalignment for walking and deep squatting activities, respectively. Experiments indicate that the exoskeleton has high compliance (0.4 and 0.1 Nm backdrive torque under unpowered and zero-torque modes, respectively), high control bandwidth (44 Hz), and high control accuracy (1.1 Nm root mean square tracking error, corresponding to 7.3% of the peak torque). This work demonstrates performance improvement compared with state-of-the-art exoskeletons.

## 1. Introduction

Powered exoskeletons have demonstrated exceptional potential for restoring the mobility of people with gait impairments [e.g., stroke survivors (Awad et al., 2017) and children affected by cerebral palsy (Lerner et al., 2017)] and power augmentation or injury prevention for healthy individuals [e.g., walking (Zhang et al., 2017; Ding et al., 2018), running (Kim et al., 2019; Sawicki et al., 2020), squatting (Yu et al., 2019), kneeling (Chen et al., 2021), etc.]. However, successful deployment of these devices in daily living is still limited by persisting challenges in the design of systems that combine effective assistance and low-profile wearability. Despite providing assistance, the exoskeleton also brings negative impacts to wearers caused by the mass, mass distribution, backdrive actuator, and wearable structure fitting, adding pressures to the human-exoskeleton contacting points and stress on tissues/bones. The net assistance performance of the exoskeleton is the assistance provided by an ideal exoskeleton, minus the negative impact of the exoskeleton on the human.

One negative effect that cannot be ignored is the misalignment of the exoskeleton with the human joints. Since exoskeletons are systems developed to work in parallel with the biological muscles, it is crucial that the robot joint remains aligned with the human joint during movement. Otherwise, the misalignment contributes to detrimental effects both on the comfort and on the effectiveness of the assistance, since it restricts the free movements of the user and deteriorates the power transmission efficiency from the actuator to the human limb (Zanotto et al., 2015; Sarkisian et al., 2021; Bulea et al., 2022; Chang et al., 2022; Chen et al., 2022).

In particular, this alignment requirement is especially challenging for the knee, which realizes sophisticated movements thanks to a compound structure consisting of two articulations, tibiofemoral and patellofemoral. There have been extensive studies that analyze the shapes of the femoral and tibial condyles and their relative movements (Weber and Weber, 1992; Pinskerova et al., 2000). More recently, accurate models have been enabled by advanced non-invasive methods relying on motion tracking of markers on the skin as well as X-ray and magnetic resonance imaging (Kordasz and Sauer, 2013). While the knee is functionally a three-dimensional joint, allowing for flexion-extension, adduction-abduction, and internal-external rotation, only its predominant movement in the sagittal plane is usually modeled for wearable robots' design. Nevertheless, its sagittal behavior is not obvious, as the axis of rotation of the femur relative to the tibia is not constant (Walker et al., 1972; Lee and Guo, 2010). In other words, their relative motion involves both rotational and translational components. Despite this, the vast majority of current exoskeletons mimic the knee joint using a 1-degree of freedom (DOF) revolute joint (Yu et al., 2019; Lee et al., 2021; Zhu et al., 2021). This fixed pin-joint design represents an oversimplification that cannot account for the complex kinematics of the knee; thus, it inevitably introduces some misalignment between the exoskeleton and the wearer's leg. Several works developed passive or tethered knee exoskeleton that can achieve self-aligning of the knee joint, which inspire the design of portable self-aligning knee exoskeleton. Kapci et al. presented a bio-joint shaped knee joint exoskeleton that can biomimic knee joint rotation during walking and sit-to-stance and provides assistance passively with a spring element (Carrozza et al., 2018; Kapci and Unal, 2018), Celebi et al. presented cable drive tethered knee exoskeleton with a planar parallel mechanism to achieve knee joint self-alignment (Celebi et al., 2013).

In addition to the alignment, other vital factors that affect the usability of a wearable robot are its mass and compliance. On the one hand, reducing the mass of the exoskeleton and moving the mass closer to the user's center of mass can reduce the weight penalty on the wearer's body (Karavas et al., 2015; Ding et al., 2018). On the other hand, it is essential to ensure a compliant behavior of the device to enable the free movement of the user. In this regard, the backdrivability (backdrive torque, smaller is better) of the actuator is usually adopted as a metric of compliance (Wensing et al., 2017; Sridar et al., 2020; Yu et al., 2020).

Recently, several solutions have been proposed for the design of a portable knee exoskeleton, and researchers leveraged various mechanisms to reproduce the human knee kinematics in knee exoskeletons. One approach exploits the introduction of additional passive DOFs the compensation for the misalignment between the human and the robotic limbs. An illustrative embodiment of this method is reported in Choi et al. (2016), where the authors developed a self-aligning joint mechanism based on four pulleys and two connecting links. The mechanism is integrated into a platform for the assistance of the whole lower limb. It takes advantage of cable transmission to remotely locate the actuation unit on the wearer's back. However, its considerable mass (14.7 kg) and the employment of a ball screw in the actuation system still limit the transparency and the compliance of the device and pose substantial challenges to its wide usability. Another method involves the use of a curved guide rail to match the biological trajectory of the instantaneous center of rotation of the knee joint (Kim et al., 2021). In this case, the power transmission is accomplished through a mechanism similar to the four-bar linkage, where it is possible to adjust the transmission ratio by changing the length of the linkages. Further, a knee exoskeleton with a rolling joint mechanism is implemented in Wang et al. (2018), where the roller diameter is designed to minimize the misalignment and a two-stage timing belt system is used to transmit the torque from the motor located at the proximal end of the thigh.

**Figure 1:**
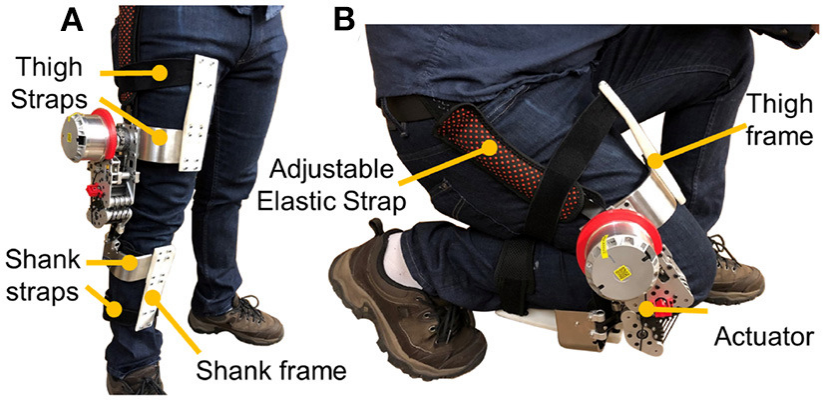
The bio-inspired design of a lightweight cable-driven knee exoskeleton aims to reduce negative design effects in terms of joint misalignment mitigation, lightweight, and high compliance. **(A)** Side view in a standing position. **(B)** Crouched down posture that demonstrates the wide range of motion of the device, being not restrictive of the wearer's free movements.

Our previous research proposed fixed-axis rotation knee and hip exoskeletons that can assist walking and squatting (Yu et al., 2019, 2020; Huang et al., 2022). The scope of our work targets to develop a portable bio-inspired knee exoskeleton that can further reduce weight penalty while ensuring mitigating misalignment and high compliance, as shown in Figure 1. It can mitigate joint misalignment by the design of rolling gears for mimicking the relative motion of the tibia and femur and can achieve high compliance by a cable-driven amplification mechanism. Simulation results indicate our design can reduce 49.3 and 71.9% maximum total misalignment for walking (θ_max_ = 60°) and deep squatting (θ_max_ = 120°) activities, respectively. Leveraging on the combination of high torque density motor and high stiffness cable-driven amplification mechanism in this work, the proposed exoskeleton is also lightweight (1.7 kg exclude 292 g batteries) and highly compliant (0.4 and 0.1 Nm backdrive torque under the unpowered and zero-torque mode, respectively), which further reduce the negative effect of knee exoskeleton to human. In addition, the proposed design also has high control bandwidth (44 Hz) and high torque tracking accuracy (1.1 Nm root mean square tracking error, 7.3% of the desired peak torque). To the best of our knowledge, this is the most lightweight portable knee exoskeleton that is designed to mitigate joint misalignment and have high compliance to minimize restriction of human nature motion.

## 2. Design requirements and solutions of bio-inspired cable-driven knee exoskeleton

One major purpose is to design a bio-inspired exoskeleton that mitigates the misalignment between the human and the device joint. In addition to misalignment mitigation, for a portable knee exoskeleton to be effectively deployable, factors like device mass, assistive torque, backdrivability (i.e., resistive torque under unpowered conditions), and control bandwidth are also paramount. An ideal knee exoskeleton should be lightweight to reduce the impact on the inertial leg properties as much as possible and minimize the energetic cost of wearing the device. On the other side, the torque delivered to the wearer should be as large as possible to provide substantial assistance. In contrast, minimum resistive torque is desirable not to constrain the free movements of the wearer. Ultimately, to quickly respond to the sudden change, the bandwidth of the exoskeleton is recommended to be greater than the maximum frequency of human walking, i.e., 3 Hz (Ji and Pachi, 2005).

Thus, the design requirements are listed in Table 1, where the assistive torque is set to 16 Nm, corresponding to 40% of the biological torque (Schmidt et al., 2017), while the backdrive torque is set to 2 Nm, i.e., 5% of the biological torque applied during human walking (Ding and Park, 2017). Our target is to design a knee exoskeleton that can mitigate joint misalignment, have high compliance, and has comparable mass (2 kg) to the advanced designs (Chen et al., 2021; Zhu et al., 2021). The final design parameters of our designed knee exoskeleton are also listed, which satisfies the design requirements. The design is discussed in detail in the following sections.

**Table 1:** Design requirements for the knee exoskeleton.

**Feature**	**Unit**	**Requirement**	**Our design**
Assistive torque	Nm	≥16	16
Bandwidth	Hz	≥3	35
Backdrive torque	Nm	≤2	0.4
Mass (no battery)	kg	≤2	1.7

To meet the above requirements, we opted for combining a high torque density actuator with a cable-driven transmission mechanism. The specification of the presented tension amplifying mechanism is shown in Table 2. The mechanical stiffness of the cable-tension amplification mechanism is 576 N/mm, and the output torque is 16 Nm.

**Table 2:** Specification of high-stiffness tension-amplification mechanism.

**Parameters**	**Value**
Gear ratio *n*	8
Stiffness − amplification ratio *n*^2^	64
Cable stiffness	9 N/mm
Mechanism stiffness	576 N/mm
Input torque	2 Nm
Output torque	16 Nm

## 3. Bio-inspired design of the cable-driven knee exoskeleton

The design of the proposed bio-inspired cable-driven knee exoskeleton involves two significant components: a bio-inspired rolling joint, and a cable-based transmission amplification mechanism. Both components are discussed in the dedicated following sub-sections. In addition, the proposed bio-inspired cable-driven actuator is connected to the thigh and shank frames. Both the thigh and shank frames contain two straps, which can help secure the exoskeleton to ensure tightness and fit well on the person, as shown in Figure 1. One adjustable elastic strap is used to provide pretension force for the exoskeleton weight bearing, avoiding potential misalignment caused by the robot slide during motions. To ensure comfort but tightness, wearers were asked to sit on the chair and relax their muscles when donning the exoskeleton.

### 3.1. Bio-inspired rolling joint design

**Figure 2:**
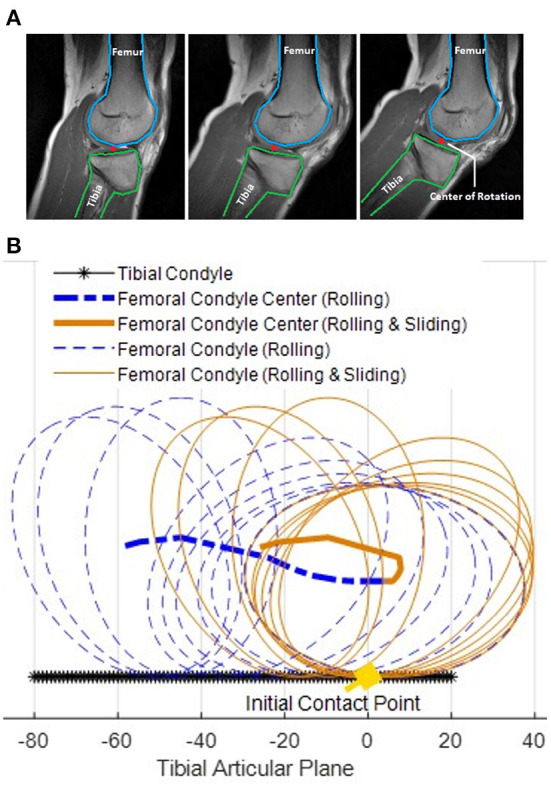
**(A)** Magnetic resonance imaging (MRI) scanned for human knee joint when the knee joint is moving from extension (left) to flexion (right). The blue and green contours indicate the femur and the tibia, respectively. The red dot represents the knee joint center of rotation. **(B)** Simulation result of femoral condyle center during −5° to 120° knee flexion movement using both pure rolling model (dotted blue lines) and rolling-sliding combined model (solid orange lines). Compared with the pure rolling femoral condyle center trajectory (bold dashed blue line), the rolling and sliding joint condyle center trajectory (bold solid orange line) allows the center of the femoral condyle to have smaller locomotion at the tibial articular plane with respect to the tibia (black line). The result indicates a non-fixed axis robot knee joint can mitigate misalignment compared with a traditional axis fixed robot knee rolling joint.

Inspired by the human knee flexion-extension motion, the proposed design leverages a rolling mechanism for the proper alignment between the human and the exoskeleton knee joints during daily activities. Unlike coaxial motion, the human knee flexion-extension motion is characterized by a non-fixed rotation center. Figure 2A shows three magnetic resonance imaging (MRI) scans, which illustrate the instantaneous center of rotation during different phases of the human knee joint flexion-extension motion. As observed from the MRI in Iwaki et al. (2000), during the knee joint flexion over −5° to 120°, the femoral moved forwards 19 mm relative to the tibia by rolling vs. sliding in the ratio of 1.7:1. Among the 19 mm movement, 15 mm of the displacement happened between 45° to 120° knee rotation, and 4 mm happened between −5° to 45° knee rotation. Compared with fixed-axis rotation, human knee joint occurs non-negligible displacement during walking (60° rotate) and squatting (120° rotate). Bioinspired by the knee rotary MRI scans, the exoskeleton knee joint should not be designed as a fixed axis but bio-mimic the rotatory trajectory of the human knee joint that combines rolling and sliding movement.

Previous studies on knee mechanics have revealed that the relative movement between the femoral and tibial condyles in the sagittal plane is essentially depictable as an ellipse-line cam mechanism (Choi et al., 2016; Wang et al., 2018; Kim et al., 2021; Sarkisian et al., 2021). The motion of the biological knee is neither pure sliding nor pure rolling, but a combination of rolling and sliding with a certain sliding ratio (Li et al., 2018). A biological knee joint model (Li et al., 2018) describes the relative motion of the femoral condyle for the tibial condyle as an ellipse rolling and sliding. The sliding ratio in this model, which is defined as the ratio between rolling sliding distances can be adjusted to emulate the complex movements in the biological knee joint. A representative biological knee joint parameter (Opensim gait2392) obtained in Kainz et al. (2017) is used in the model, as shown in Table 3.

**Table 3:** Physical parameters of the lower limb in the model.

**Parameter**	**Value**
Femur length (mm)	39
Tibia length (mm)	36
Tibia mass (kg)	3.6
Femur inertia (kgm^2^)	0.14
Tibia inertia (kgm^2^)	0.05
Semi-major axis of femoral condyle (mm)	33.6
Semi-minor axis of femoral condyle (mm)	23

The simulation results of comparing the trajectories of a pure rolling model (dotted blue ellipses) and a rolling and sliding model (solid orange ellipses) during knee flexion motion from −5° to 120° knee angle are shown in Figure 2B. The solid orange ellipses represent the rolling and sliding model adopted in our simulation framework, and the dotted blue ellipses represent the rolling-only model. At the initial position, −5°, the orange and blue ellipses overlap and share the same initial contact point, while their trajectories diverge as the knee angle increases. Compared with pure rolling, the combination of rolling and sliding allows the center of the femoral condyle to have smaller locomotion, which is more similar to the observed data in Pinskerova et al. (2000).

**Figure 3:**
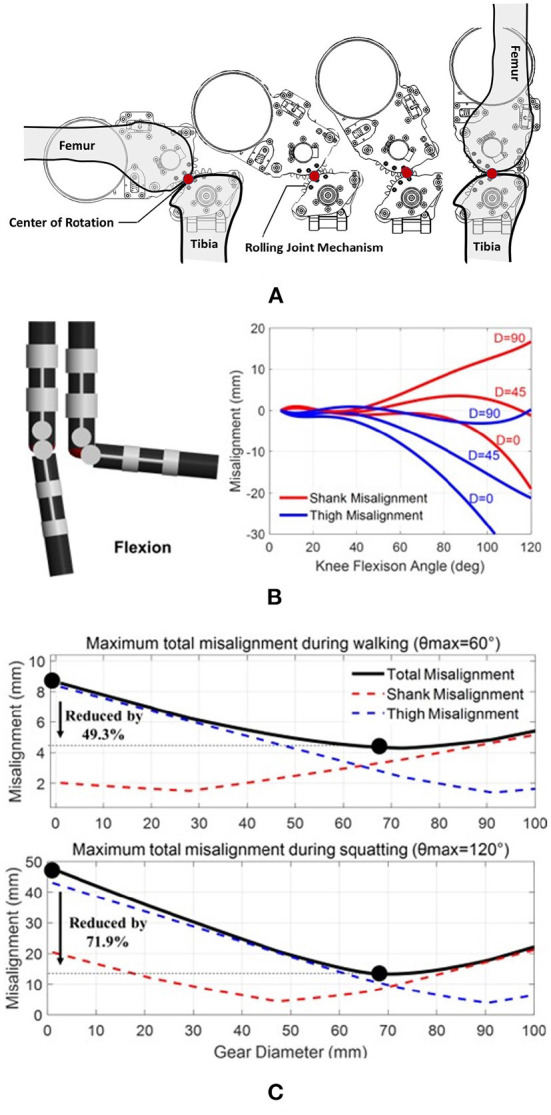
**(A)** The bioinspired rolling-gear based knee joint mechanism can provide equivalent rolling and sliding motion during knee joint movement. Compared with traditional fi*x*-axis rotation robot joint designs, the design can mitigate joint misalignment. The non-fixed rotation center is defined by the contact point between the two gears; **(B)** Simulation of the misalignment effect for the rolling joint design. The red and the blue lines represent the misalignment functions at the shank and the thigh during the knee flexion motion, respectively; **(C)** Joint misalignment reduction simulation results during walking (θ_max_ = 60°) and deep squatting (θ_max_ = 120°).

To mimic the translation of the rotation center and ensure a proper alignment between the exoskeleton and the human leg, we designed a rolling-gear based knee joint mechanism with a non-fixed rotation center, as shown in Figure 3A. The design principle of this rolling gear is similar to Wang et al. (2018). It can provide equivalent rolling and sliding motion during knee joint movement. In this case, the center of rotation is defined by the contact point between the engaging gears, thus the diameter of the gears plays a key role in the misalignment mitigation. To quantify the misalignment and guide the bioinspired knee joint design, a thigh-knee-shank model is built based on Simscape Multibody and run by MATLAB/Simulink (Li et al., 2018). The simulation results show a smaller gear diameter (e.g., D = 0 mm) will increase both thigh and shank misalignment, while larger gear diameters (e.g., D = 90 mm) will increase shank misalignment, as shown in Figure 3B. A misalignment factor (Wang et al., 2018) is defined to evaluate the overall misalignment on both thigh and shank, as shown in Equation (1):


(1) $$\begin{array}{l} \Phi \left(\text{D}\right)=\underset{0\leq \theta \leq \theta_{\text{max}}}{\text{max}}\sqrt{|T\left(\theta ,x\right){|}^{2}+|S\left(\theta ,x\right){|}^{2}} \end{array}$$

Where θ_*max*_ represent the maximum knee flexion angle considered, *T*(θ, *x*) and *S*(θ, *x*) respectively represent the amount of thigh and shank misalignment *x*, determined by knee angle θ and roller diameter D.

Considering the displacements between the exoskeleton frames and the thigh or shank as metrics of the misalignment, we found out the gears' diameter that D = 68 mm is a suitable gear diameter for our design, as shown in Figure 3C. Simulation results show that compared with fixed axis rotation (D = 0 mm), the rolling gear design (D = 68 mm) can mitigate 49.3 and 71.9% misalignment factor Φ for walking (θmax = 60°) and deep squatting (θmax = 120°), respectively.

### 3.2. High stiffness cable-tension amplification mechanism

Compared to gear transmission, traditional cable-driven transmission has the advantages of lightweight and low profile but has low stiffness due to cable elasticity. The use of cable transmission will reduce system stiffness to reduce system control bandwidth and slow response to the control command. This manuscript proposed a design of high stiffness cable-tension amplification mechanism that considers both lightweight and high stiffness transmission. It offers three primary advantages: (1) compared to a conventional gearbox, the cable tension amplification mechanism is more lightweight and low-profile; (2) the design positions the actuator closer to the wearer's proximal side, which can reduce the device weight penalty; (3) it ensures high bandwidth torque control thanks to the high stiffness of the cable transmission.

**Figure 4:**
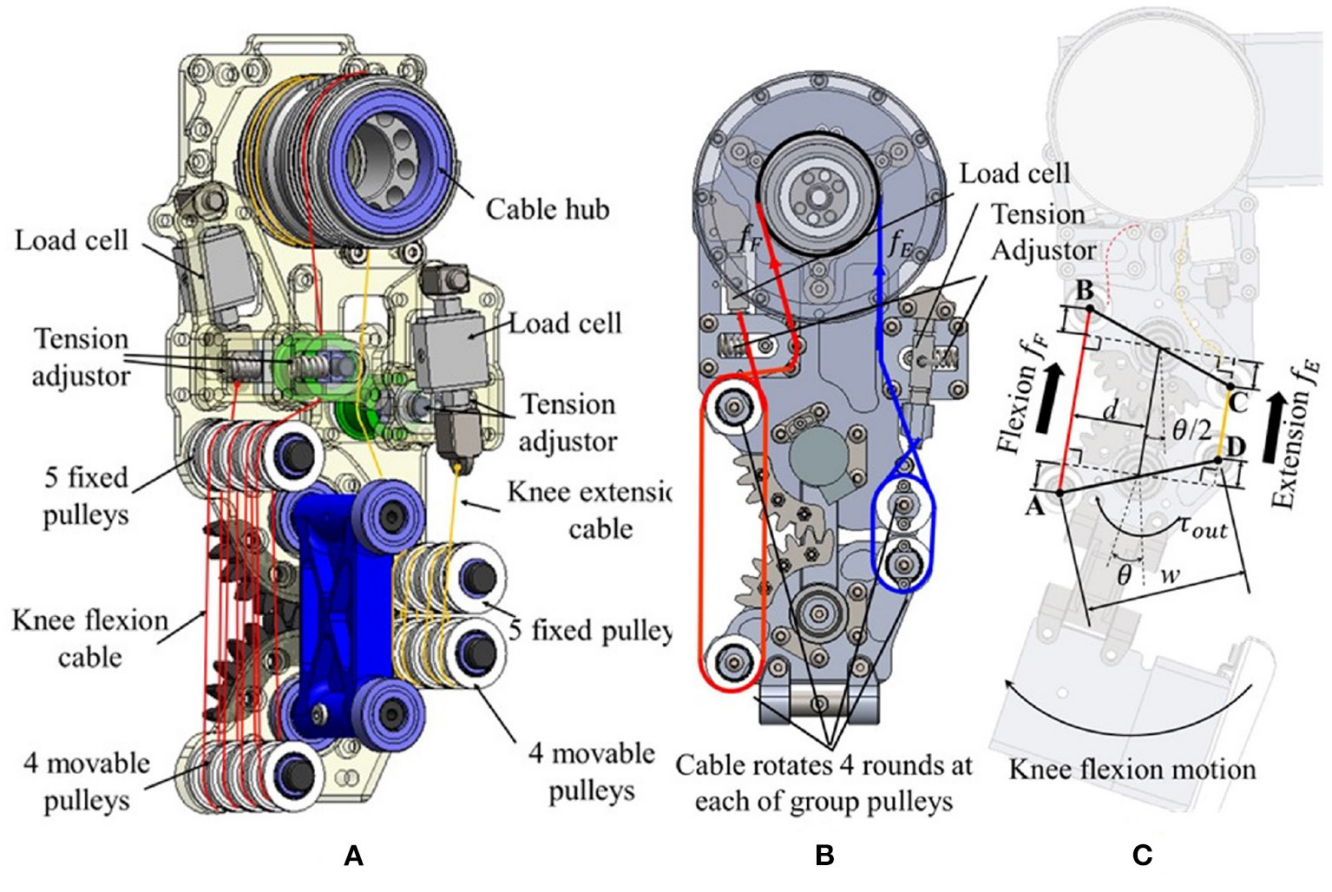
The detailed structure of the knee exoskeleton. **(A)** The flexion cable (red line) and extension cable (yellow line) is driven by a cable hub and pass through fixed (upper) and movable (lower) pulleys used to realize the tension-amplification mechanism. **(B)** Cable routing, force sensing, and tension adjuster. **(C)** Relationship between the cable motion and joint angle. High stiffness cable-tension amplification mechanism. The tension-amplification mechanism realizes the function of the speed reduction mechanism with a gear ratio of 8 and the resultant stiffness is 64 times the stiffness of the cable, see Equation (8).

The detailed schematic of the cable-driven knee exoskeleton with the tension amplification mechanism is illustrated in Figure 4A. Two cables are used to achieve knee joint flexion (red cable) and extension (yellow cable) *via* bidirectional control of a single actuator. The actuator composes a customized high torque density motor assembled in a cable hub. Starting from the cable hub, each cable goes through a guiding pulley (in green), turn around some fixed (upper) and moveable (lower) pulleys, which serve as tension amplification mechanism, as described in the next section, and has the other end fixed to a load cell (FSH01670, FUTEK, 50 lb. capacity). All the pulleys are freewheeling pulleys with bearings. The diameters of the fixed and moveable pulleys are specifically designed to reduce the slope angle of the cables between them (less than 5 degrees). In this way, the length change of the cable in both directions is allowed to maintain constant. To compensate for the loosened of the cable due to friction and maintain high stiffness transmission for both extension and flexion movements, a pair of auto-tension adjustors are attached with the guiding pulleys and fitted into sliding slots. Each adjuster is made up of two compression springs which are located at the two ends of the cable, as shown in Figure 4A. The spring (LC029C01S, Lee Spring^®^) has a 9.25 mm free length, 4.32 mm solid length, and 4,886 N/m spring rate, which can allow the adjuster to provide a maximum of 50 N (25 N per spring) pretension force for the cable transmission mechanism. Both the design of pulleys and tension adjustors work together to ensure the stability and functionality of our proposed cable drive mechanism.

The base frame of the exoskeleton is made of Aluminum 7075 T6 with strong yield stress (503 MPa) and low mass density (2,810 kg/m^3^). The cable is a 2 mm Mini-V double braided Vectran cable (spun liquid crystal polymer, New England Ropes), with negligible cold creep and a breaking strength of over 1,200 N, ensuring a safety factor of over 1.6 to support the maximum output tension.

As mentioned above, the tension-amplification mechanism comprises fixed and movable pulleys. It transmits the power from the motor (positioned on the thigh) to the knee joint and amplifies torque. The routing of the cables is shown in Figures 4B,C shows the geometric relationship between the cables' lengths and the joint angle. The centers of the movable and fixed pulleys (on both flexion and extension sides) form an isosceles trapezoid ABCD during the knee joint rotation. The two bases of the isosceles trapezoid are the flexion-side and extension-side cables between the centers of the relative pulleys, respectively, and the legs *w* that denote the lengths of the bases. Therefore, when the knee joint rotates, the total change of the flexion δ_*f*_ and extension cables δ_*e*_, should be the same (δ), as shown in the following equation


(2) $$\begin{array}{l} \delta =nw\sin\frac{\theta }{2} \end{array}$$

where *n* = 8 is the number of turns of the cables around the fixed and moveable pulleys on both flexion and extension sides, and *w* is the distance between the centers of the flexion side pulleys and the extension side pulleys (i.e., legs of the trapezoid ABCD), and θ is the knee joint angle.

Assuming that the friction between pulleys (2%) and cables can be neglected, the output cable force *f*_*out*_ and torque τ_*out*_ at the joint is derived as


(3) $$\begin{array}{l} f_{in}=f_{E}-f_{F} \end{array}$$

(4) $$\begin{array}{l} f_{out}=nf_{in} \end{array}$$

(5) $$\begin{array}{l} d=\frac{w}{2}\cos\theta \end{array}$$

(6) $$\begin{array}{l} \tau_{in}=df_{in}=\frac{w\cos\theta f_{in}}{2} \end{array}$$

(7) $$\begin{array}{l} \tau_{out}=n\tau_{in} \end{array}$$

Where *f*_*in*_, *f*_*out*_, τ_*in*_, and τ_*out*_ denote the input and output cable force and joint torque, respectively, *d* is the moment arm, and *f*_*F*_ and *f*_*E*_ are the flexion and the extension cable tensions, respectively.

Therefore, this cable-driven system functions as a transmission mechanism with gear ratio *n*, which amplifies the torque and reduces the rotational speed by a factor of *n* relative to the input quantities from the motor. Moreover, the resultant stiffness of the mechanism is


(8) $$\begin{array}{l} k_{out}=\frac{f_{out}}{x_{out}}=\frac{nf_{in}}{x_{in}/n}=n^{2}\frac{f_{in}}{x_{in}}=n^{2}k_{in}, \end{array}$$

where *k*_*out*_ is the stiffness of the knee joint, while *k*_*in*_ = *f*_*in*_/*x*_*in*_ is the cable stiffness; *x*_*in*_ is the elongation of the cable; *x*_*out*_ is the elongation of the distance between a pulley pair. Therefore, the stiffness amplification factor of the mechanism is *n*^2^. In the current design, *n* is equal to 8, denoting that the output torque is amplified by a factor of 8 while the output stiffness is amplified by a factor of 64.

## 4. Modeling of physical human-exoskeleton interaction

**Figure 5:**
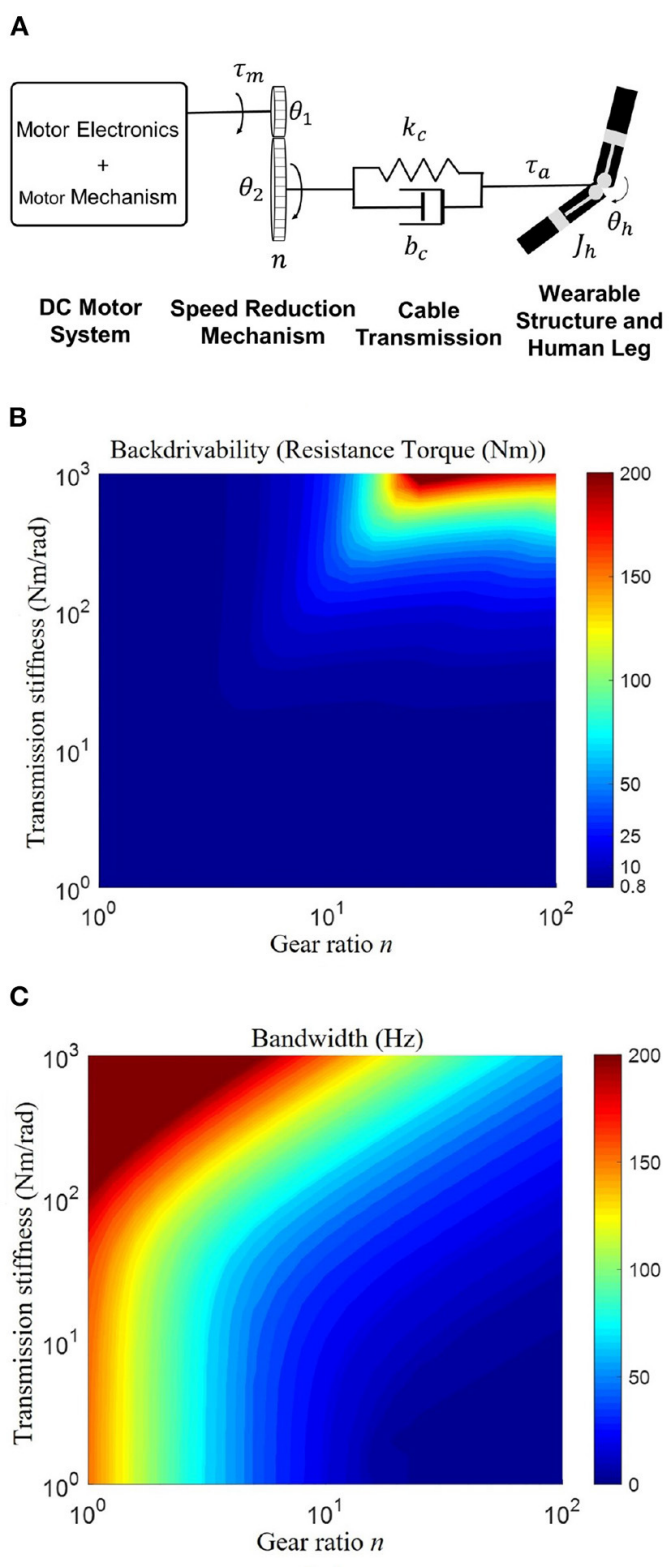
**(A)** The framework of the human-exoskeleton interaction model. This model consists of four modules: motor system, speed reduction mechanism, cable transmission, and wearable structures attached to the human. θ_1_ and θ_2_ denote the rotation angles of the input gear and output gear, respectively. *n*, speed reduction ratio; *k*_*c*_, transmission stiffness; *b*_*c*_, transmission damping; *J*_*h*_, joint inertia of the shank orthosis and human shank; θ_*h*_, knee angle; τ_*m*_, motor torque; τ_*a*_, exoskeleton actual torque. **(B,C)** Influence of gear ratio and transmission stiffness on backdrivability and control bandwidth. **(B)** Illustrates the high backdrivability is ensured by either high transmission stiffness or small gear ratio; **(C)** Shows the high bandwidth requires both high transmission stiffness and small gear ratio. Therefore, both a high transmission stiffness and a small gear ratio are necessary to ensure high backdrivability and high bandwidth.

The tradeoff between critical properties like bandwidth and compliance (backdrivability) is one of the main challenges in actuator design (Zhu et al., 2022). To theoretically evaluate the influence of the design paraments on these metrics, a human-exoskeleton interaction model is used to analyze the actuation performance. As shown in Figure 5A, this model considers various components, including the motor system, the speed reduction gearbox, the transmission, and the wearable structure.

The speed reduction mechanism can be cable-driven, a gearbox, belt or chain transmission, etc. Among the several options, the cable-driven transmission allows for lightweight, low output inertia, low friction, low backlash, and high stiffness, thus representing a good solution for high backdrivability and torque control bandwidth.

The speed reduction ratio determines the inertia ratio of the loading side and motor side. Additionally, a higher gear ratio will generate higher inertial and frictional forces on the loading side, which affects the backdrivability and bandwidth. Therefore, we will focus on the influence of the reduction ratio and transmission stiffness on the backdrivability, and bandwidth based on the human-exoskeleton interaction model.

In this case, we are interested in expressing the dependency of mechanical backdrivability and control bandwidth on our design parameters, which are the gear ratio *n* and the transmission stiffness *k*_*c*_. To analyze the sensitivity of the parameters of backdrivability and bandwidth, we derived the transfer function of backdrivability, and control bandwidth based on the modeling of human-exoskeleton interaction.

For backdrivability analysis, the transfer function is shown in Equation (9).


$$\begin{array}{ll} \; & G_{backdrivability}= \end{array}$$

(9) $$\begin{array}{l} \frac{\tau_{a}\left(s\right)}{\theta_{h}\left(s\right)}=\frac{-n^{2}s\left(b_{c}s+k_{c}\right)\left(J_{m}R\text{s}+Rb_{m}+K_{b}K_{t}\right)}{n^{2}J_{m}Rs^{2}+\left(n^{2}Rb_{m}+n^{2}K_{b}K_{t}+Rb_{c}\right)s+Rk_{c}} \end{array}$$

Where *n* is gear ratio, *k*_*c*_ is cable transmission stiffness, *R* is winding resistance; *b*_*m*_ is motor damping; *b*_*c*_ is cable transmission damping; *K*_*b*_ is motor back EMF constant; *K*_*t*_ is motor torque constant; *J*_*m*_ is motor rotor inertia.

We test the resistive force of the exoskeleton joint to evaluate the backdrivability when the motor is unpowered and the joint rotates a total of 10 degrees at a maximum frequency of 10 Hz, which exceeds the maximal rotation frequency of the human joint (Zawadzki and Siemieński, 2010). A 0–10 Hz chirp signal with an amplitude of 10 degrees was used to command the input signal θ_*h*_ (knee joint angle). The output signal, τ_*a*_, is the actual torque applied on the human shank.

For bandwidth analysis, a standard PID torque controller is implemented in the simulation, whose proportional, integral, and derivative gains are *k*_*p*_, *k*_*i*_, and *k*_*d*_, respectively. The input signal is the reference torque τ_*r*_ and the output signal is the actual torque τ_*a*_ applied on the human shank. The resulting transfer function is:


(10) $$\begin{array}{l} G_{bandwidth}=\frac{\tau_{a}\left(s\right)}{\tau_{r}\left(s\right)}=\frac{nk_{t}\left(b_{c}s+k_{c}\right)}{n^{2}\left(J_{m}R+Rb_{m}+K_{b}K_{t}\right)s^{2}+Rb_{c}s+Rk_{c}} \end{array}$$

The simulation is performed by fixing the human shank [θ_*h*_(*s*) = 0] and testing the bandwidth of the torque control.

Table 4 lists the motor and PID parameters used for the modeling. To simplify the simulation model, the terms with inductor are ignored and doesn't appear in both Equations (9) and (10) due to the inductance term of the motor being low.

**Table 4:** Specification of high-stiffness tension-amplification mechanism.

**Parameter**	**Symbol**	**Value**	**Unit**
Motor resistance	R	2.28	Ω
Motor inductance	*L*	2.5E-3	H
Supply voltage	*V*	48	Voltage
Torque constant	*k*_*t*_	0.217	Nm/A
Back emf constant	*k*_*b*_	0.217	V/rad
Motor friction coefficient	*b*_*m*_	0.001	Nms/rad
Rotor inertia	*J*_*m*_	$3\times 10{-}^{3}d_{m^{3}}$	Kgm^2^
Reduction ratio	*n*	1~100	-
Transmission stiffness	*k*_*c*_	1~10,000	Nm/rad
Transmission damping	*b*_*c*_	0.001	Nms/rad
Human leg inertia	*J*_*h*_	0.8	Kgm^2^
P gain	*k*_*p*_	200	-
I gain	*k*_*i*_	0	-
D gain	*k*_*d*_	10	-

The maximum resistive force and control bandwidth in the simulation with various gear ratios and stiffnesses are shown in Figures 5B,C, respectively. It depicts the simulated maximum resistive force and controller bandwidth as functions of the gear ratio and transmission stiffness. It reveals that high backdrivability (note that high backdrivability corresponds to low resistive forces and high compliance) can be achieved for either a small gear ratio or low transmission stiffness. On the one hand, reducing the transmission stiffness also implies a reduction in the bandwidth of the torque controller, thus a transmission with high stiffness is desirable. On the other hand, the bandwidth increases as the gear ratio decreases, meaning that the gear ratio should be as small as possible.

In conclusion, these results demonstrate that the combination of high-stiffness transmission and small gear ratio provides a viable solution for designing an exoskeleton with high backdrivability and high control bandwidth in human assistance. Inspired by the modeling and analysis, our designed cable-driven amplification mechanism has an 8:1 torque amplification with 576 Nm/m stiffness, as shown in Table 2.

## 5. Actuation and control

**Figure 6:**
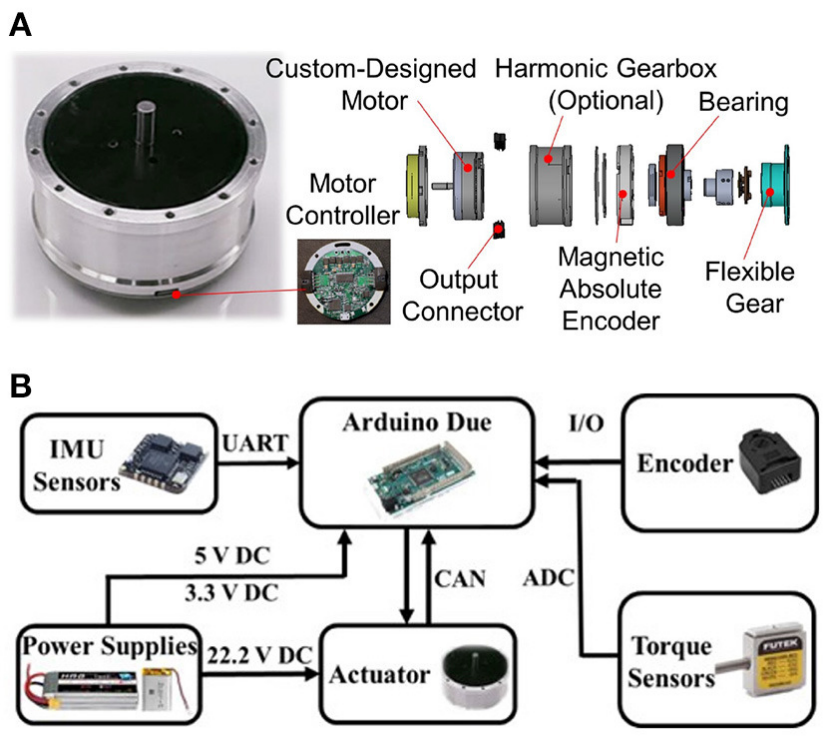
**(A)** Overview and exploded view of the custom-designed fully-integrated, lightweight, direct-drive actuator used in the design of the knee exoskeleton **(B)**.

To minimize the reduction ratio while also providing effective torque assistance, a high torque actuator is required. For the actuation, a customized, fully integrated direct-drive actuator was designed to accommodate the volume and mass requirements for a portable knee exoskeleton, as shown in Figure 6A. The actuator mass is 484 g and includes a high torque density brushless DC electric motor (BLDC motor), a 14-bit magnetic encoder, a 10–60 V wide range input driver, and a motor controller. Quasi direct-drive (QDD) actuation (Lim et al., 2015) is a new paradigm of robot actuation design that aims at increasing torque density by minimizing the transmission ratio and maximizing the torque-to-mass ratio of the motor. This actuation strategy has been recently studied for legged robots (Wensing et al., 2017) and exoskeletons (Sulzer et al., 2009; Lim et al., 2015; Lee et al., 2017; Yang et al., 2019; Yu et al., 2020). The portable exoskeleton with a quasi direct drive actuator can have both high compliance and high control bandwidth at the same time (Yu et al., 2020). Thanks to the application of a high torque density smart actuator (Yu et al., 2020; Zhu et al., 2021), our customized motor mass is only 174 g, and under 24 V voltage can reach 2,000 RPM speed and provide a nominal torque of 0.67 Nm (peak torque over 2 Nm). As a means of comparison, the corresponding power and torque densities are more than 10 and 5 times larger than conventional Maxon EC flat90 series motors, respectively (Yu et al., 2019).

Overall, by exploiting the high torque density of the actuator and the cable-driven mechanism with an 8:1 transmission ratio, the knee exoskeleton can offer up to 16 Nm output torque with reduced impedance and enhanced backdrivability, to the benefit of safety and responsiveness in the human-robot interaction.

The electrical system and control hardware of the exoskeleton are shown in Figure 6B. The electrical system and the motor are powered by a 3.7 V (1,100 mAh, 22 g) and a 22.2 V (1,800 mAh, 270 g) lithium battery, respectively. A DC-DC regulation model is used to boost the 3.7 V voltage to 5 V to power the microcontroller, IMUs, and encoder. The control hardware accomplishes the functions of low-level motor control, high-level torque control, sensor signal measurement, data communication, and power management. The local motor controller relies on a motor driver and a microcontroller (DSP TMS320F28335, Texas Instrument, Inc.) to measure the motor status and implement current, velocity, or position control. A CAN bus is used to connect the motor controller to a high-level controller for real-time data communication. The high-level torque control runs on an Arduino Due microcontroller and acquires real-time measurements from the sensors, including the wearer's lower-limb posture (left thigh, right thigh, left shank, and right shank) from four IMU sensors (HI219M, HiPNUC, Inc.), the knee joint rotation angle from an encoder (HEDM-5500#B06, AVAGO Technologies, Inc.), and the tensile forces of the transmission cables from the two loadcells (LRM200, Futek, Inc.).

**Figure 7:**
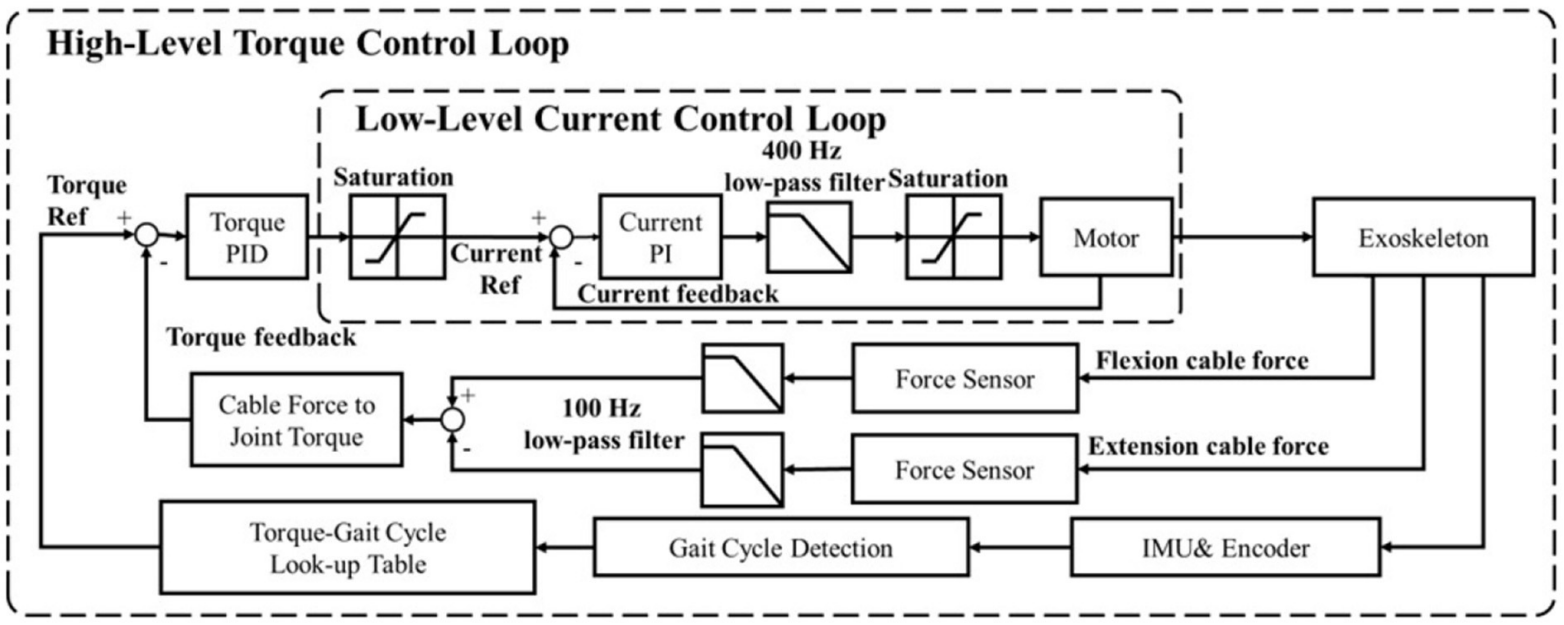
Block diagram of the control architecture. The motor low-level controller performs current PI control by using actuator integrated sensors for feedback, while the high-level torque PID control uses two force sensors to obtain joint torque feedback.

The torque control architecture is composed of the low-level motor current controller and the high-level torque controller, updated at 10 and 1 kHz frequencies, respectively, as shown in Figure 7. The output assistive torque is calculated from loadcells' measurements and fed back to the high-level torque control loop. In the ideal and low-speed conditions, the system output torque is proportional to the motor current. While under dynamic and high-speed conditions, non-linear damper and inertia terms will increase the steady-state error of the system. To mitigate steady-state error, a PID controller is implemented. The integral (I) term can reduce the steady-state error and the derivative (D) term can smooth the curve and allow the proportional (P) term to continue to increase which can further reduce the steady-state error.

A strategy that can control the assistive force using motor position control is adapted from Quinlivan et al. (2017) to model the relationship between force and position. The benefit of this strategy is that the motor position control loop has more excellent stability, and the system does not require additional torque or force sensors. On the other side, the drawback is that the actual assistive force will be less accurate due to the inherent difficulty of modeling this position-force relationship in a non-linear mechanical system. In our case, the direct measurement of the cables' tensions is able to guarantee the accuracy of the output assistive torque through a compact and lightweight sensing system, but the limitation is that the bandwidth of the output torque control must be lower than the current control method, as stated in Lv and Gregg (2017). The exoskeleton design presented in this paper uses a high torque density motor and low gear ratio in place of a traditional serial elastic actuator (high ratio gear with elastic components) to compensate for the potential control bandwidth loss. This system can thus improve the controller bandwidth while still retaining the performance benefits of improved human safety and backdrivability.

## 6. Performance evaluation

Both benchtop tests and human walking experiments are used to investigate the joint alignment, mechanical impedance (compliance), and control performance of the knee exoskeleton. They involve the evaluations of the bandwidth, the backdrivability in unpowered conditions and zero-torque control mode, and the torque tracking performance. All subjects signed an informed consent form with approval from the Institutional Review Board.

### 6.1. Joint misalignment measurement

**Figure 8:**
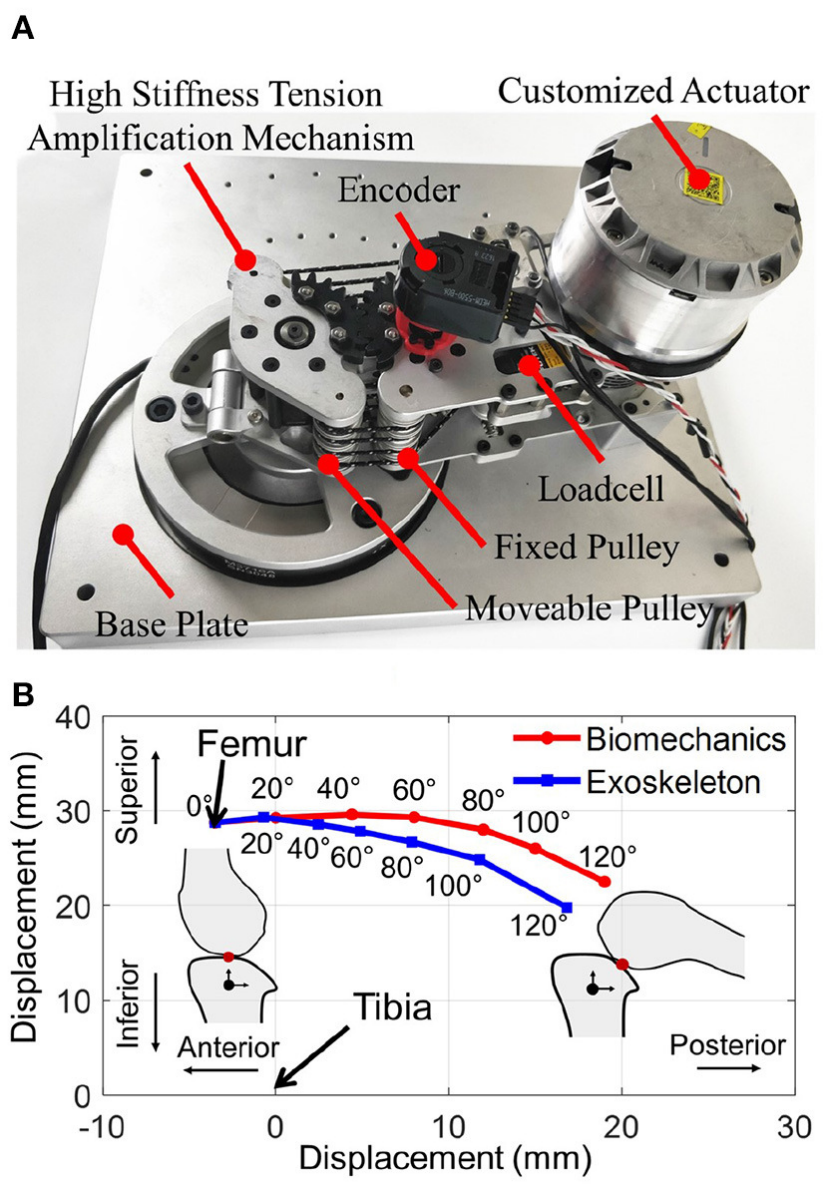
**(A)** Bench test platform that fixes the thigh and shank links of the joint mechanism; **(B)** The knee center of rotation displacement result shows our proposed rolling gear based knee joint can reduce joint misalignment and biomimic the rotation trajectory of the biomechanics of the human knee joint.

To evaluate the joint misalignment reduction performance, a test platform was built to fix the thigh and shank links of the joint mechanism, as shown in Figure 8A. We controlled the motor position and slightly rotate the knee joint from 0 to 120° flexion and record the video to measure the center of rotation of the knee joint. The device's center of rotation is plotted and compared with human biomechanics of the knee joint (Etoundi et al., 2021), as shown in Figure 8B. The result shows the misalignment between the device's center of rotation and human biomechanics of the knee joint is 0.7, 2.2, 3.5, 4.3, and 3.4 mm when the knee joint rotates 20°, 40°, 60°, 80°, 100°, 120°, respectively. The maximum misalignment happened at 100° knee joint rotation. The average and standard deviation of the misalignment between 0 and 100° is 2.52 ± 1.62 mm. The result illustrated that compared with fixed axis rotation knee exoskeleton design, the proposed rolling gear joint design can bio-mimic human knee joint rotation and help to reduce knee joint misalignment.

### 6.2. Torque control bandwidth

**Figure 9:**
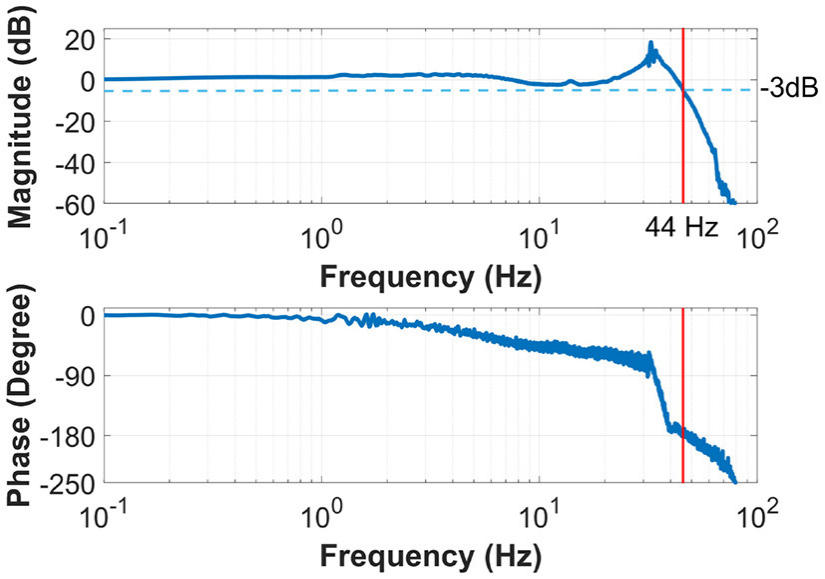
The Bode plot of the system shows a torque control bandwidth of 44 Hz, much higher than the frequency required for human walking.

Frequency analysis of the torque control strategy is carried out to analyze the torque control performance. The torque reference serves as the input signal generated by a 0–100 Hz chirp function with 5 Nm amplitude. The Bode plot is reported in Figure 9, which shows a bandwidth of 44 Hz. This bandwidth is much higher than the requirements for human walking, demonstrating that the actuator can potentially be used for quick response movements such as human running and fall risk prevention. Hence, the bandwidth of this torque control approach is more than sufficient for the assistance of human walking.

### 6.3. Backdrivability in unpowered condition

**Figure 10:**
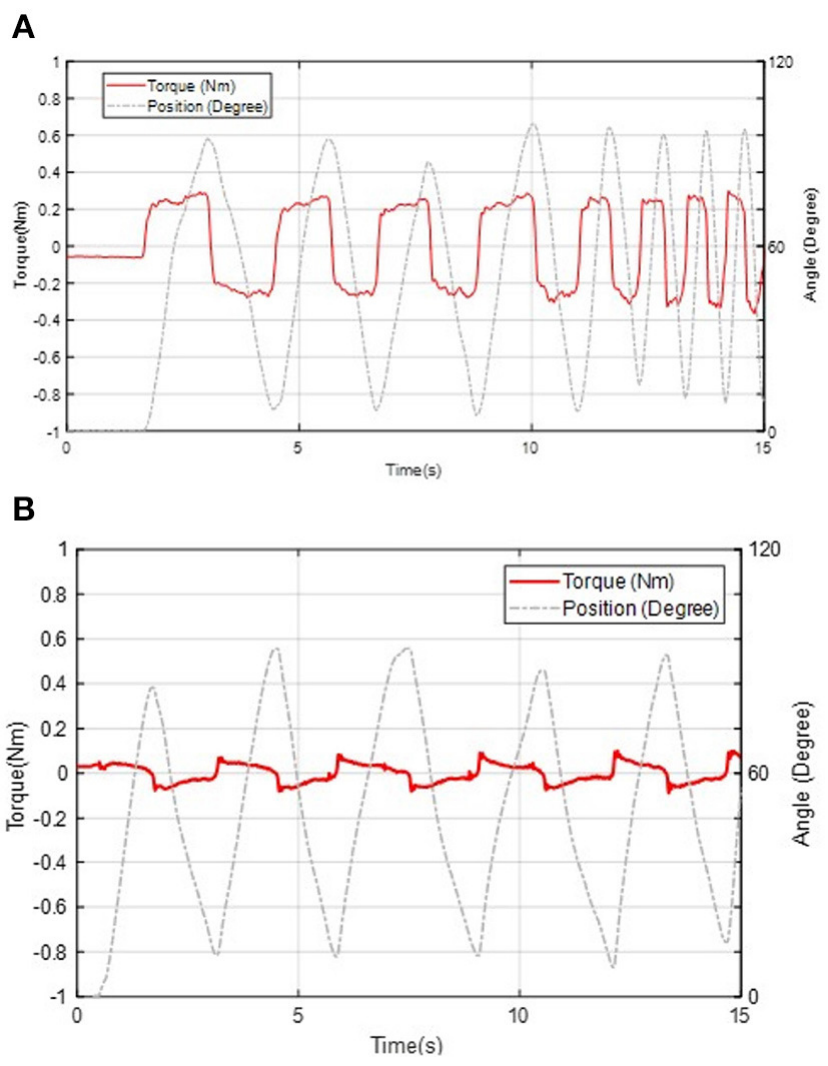
Backdrivability performance of the knee exoskeleton in unpowered mode **(A)** and zero-torque control mode **(B)**.

To demonstrate the compliance of the exoskeleton, we tested the backdrive torque in unpowered mode. One able-bodied subject wore the unpowered exoskeleton and rotate the knee joint from 0° to 90° at a cadence of 1–2 Hz. Because the cable-tension amplification transmission results in a low-gear ratio, low friction, and low backlash. It generates a low impedance mechanical system with a maximum mechanical resistive torque of approximately 0.4 Nm under unpowered conditions, shown in Figure 10A. The absolute average resistive torque during the test is 0.21 Nm.

### 6.4. Backdrivability in zero-torque control

Zero torque control can further increase exoskeleton compliance. In this experiment, the power of the actuator system is turned on and the subject moves forward and backward to test the performance of the zero-torque controller. By implementing the controller shown in Figure 10B, the maximum resistance amplitude decreases to 0.1 Nm, which is equivalent to adding 60 g mass to the center of mass of the shank when rotating the knee joint. The absolute average resistive torque during the test is 0.04 Nm. Therefore, the energy consumption caused by the mechanical impedance of the exoskeleton is negligible during zero assistive conditions.

### 6.5. Torque tracking

**Figure 11:**
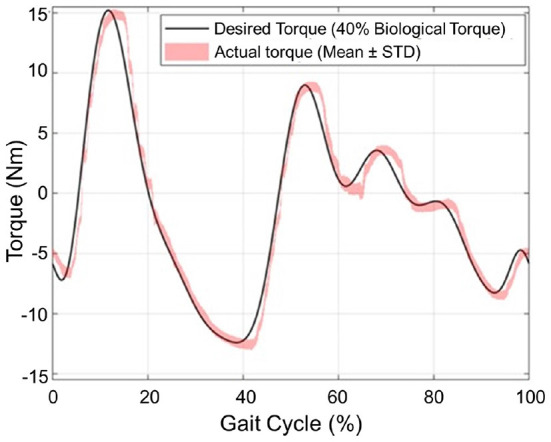
Tracking torque performance of the 16 Nm peak torque, which is about 40% knee torque assistance required during human walking.

To demonstrate the system functionality and torque assistance performance, a walking assistance torque tracking experiment for the 16 Nm peak torque profile of the knee movement (equivalent to 40% assistance torque for 80 kg subject walking on 1.25 m/s; Grimmer and Seyfarth, 2014) was performed. Three subjects (Age: 29.5 ± 4.8 years; Height: 1.76 ± 4.19 m and Mass: 76.1 ± 10.1 kg) wore the exoskeleton and walked on a treadmill. All subjects signed an informed consent form approved by the Institutional Review Board (IRB) of the North Carolina State University (IRB File #24675). The motion information of thigh and shank, acquired from IMUs and encoder, is used to estimate the phase of the gait cycle (Ding et al., 2018). We obtained the human knee joint biological torque profile from Opensim open-source model, and use this torque profile to provide 40% knee assistance in real-time during the walking experiment. The torque tracking results are shown in Figure 11. The RMS error between the desired reference and actual torque trajectory in a total of 30 strides from three subjects (10 strides from each subject) is 1.1 Nm, corresponding to about 7.3% of the maximum reference torque. This demonstrates that the torque controller can adequately track the torque profile to assist human walking.

## 7. Discussion and conclusion

To mitigate the side effect of the knee exoskeleton design caused by joint misalignment, weight penalty, and low compliance (backdrivability), the contribution of this paper is to propose the design of a portable knee exoskeleton that is self-aligning, lightweight, and high compliance. The bio-inspired design of the rolling joint mechanism can mitigate knee joint misalignment and the design of a high stiffness cable-driven transmission system can ensure high stiffness, and high compliance, and reduce mass and weight penalty by replacing the traditional gearbox and moving the actuator closer to the wearer's center of mass. In particular, the mass, output torque, backdrivability, and bandwidth have been well balanced to satisfy the needs for walking assistance. Simulation results indicate our design can reduce 49.3 and 71.9% maximum total misalignment for walking (θmax = 60°) and deep squatting (θmax = 120°) activities, respectively. The total mass of the exoskeleton is 1.7 kg (without battery) and it can generate 16 Nm peak torque, corresponding to 40% of the average knee biological torque during walking. The joint misalignment measurement result shows, compared with fixed axis rotation knee exoskeleton design, the proposed rolling gear joint design can bio-mimic human knee joint rotation and help to reduce knee joint misalignment. Both the benchtop and the three healthy subjects involved in the experiment show that the robot exhibits only 0.4 Nm resistance torque in unpowered conditions and 0.1 Nm in zero-torque control mode. The proposed design also has high control bandwidth (44 Hz) and high torque tracking accuracy (1.1 Nm root mean square tracking error, 7.3% of the desired peak torque).

Based on these results, the designed exoskeleton minimized the side effects in terms of mitigating joint misalignment, lightweight, and high compliance, which has the potential to provide beneficial assistance in not only the lab and clinical setting but also in the real-world application scenarios. The limitation of this paper is not evaluated the effectiveness of the subject in terms of human biomechanics, and also does not focus on protocol design to ensure consistent fitting. Only three healthy subjects are tested to evaluate the performance of the design. Further evaluation on a group of subjects for multiple activities assistance is needed to assess how this device can affect biomechanical and physiological measures and prepare survey forms to ask participants to ensure consistent fitting. Comparison between the proposed rolling joint knee exoskeleton design with traditional fixed axis design will be investigated as well.

## Data availability statement

The raw data supporting the conclusions of this article will be made available by the authors, without undue reservation.

## Ethics statement

The studies involving human participants were reviewed and approved by North Carolina State University, Raleigh, NC IRB File #24675, and The City College of New York, CUNY, New York, NY IRB File #2018-0885. The patients/participants provided their written informed consent to participate in this study.

## Author contributions

HS first proposed the research idea and approach of the paper and provided the project guidance. The mechatronics design, development, and fabrication of the robotic exoskeleton were done by SY, T-HH, SZ, AD, and TW. The code implementation, development, experiment testing, and data analysis were done by SY and T-HH. The first draft of the manuscript was written by SY and T-HH. SZ, AD, TW, QF, and HS provided valuable suggestions and feedback on the draft. QF and HS made significant revisions to the final paper. All authors contributed to the article and approved the submitted version.

## Funding

This work was in part supported by the National Science Foundation CAREER award CMMI 1944655, NIH R01EB029765, NIDILRR 90DPGE0011, and NIDILRR Switzer Distinguished Fellowship SFGE22000372. Any opinions, findings, and conclusions, or recommendations expressed in this material are those of the author(s) and do not necessarily reflect the views of the funding organizations.

## Conflict of interest

The authors declare that the research was conducted in the absence of any commercial or financial relationships that could be construed as a potential conflict of interest.

## Publisher's note

All claims expressed in this article are solely those of the authors and do not necessarily represent those of their affiliated organizations, or those of the publisher, the editors and the reviewers. Any product that may be evaluated in this article, or claim that may be made by its manufacturer, is not guaranteed or endorsed by the publisher.
